# Comment on “Maternal-fetal outcomes of women with hypertensive disorders of pregnancy”

**DOI:** 10.1590/1806-9282.20231285

**Published:** 2024-03-04

**Authors:** Xi Yang, Gang Wang

**Affiliations:** 1 Shiyan Maternal and Child Health Hospital, Department of Women’s Health – Shiyan, China.; 2 Shiyan Maternal and Child Health Hospital, Department of Medical – Shiyan, China.

Dear Editor,

We read with great interest the recent study^1^ investigating the maternal-fetal outcomes of women with hypertensive disorders during pregnancy. This study offers valuable insights into the impact of these disorders on both expectant mothers and their unborn children. The research meticulously examined a cohort of pregnant women grappling with hypertensive disorders, providing a comprehensive analysis of the associated risks and potential complications. It elucidated the complex ways in which these disorders can affect maternal health, underscoring the necessity for heightened monitoring and care during pregnancy. Furthermore, the study^1^ demonstrates the consequences for fetal outcomes, presenting a thorough overview of the potential challenges that may arise. It explored factors such as prematurity birth <34 weeks, NICU admission, and perinatal mortality, offering a holistic understanding of these disorders. The findings of this study hold promise for informing clinical practices and interventions for women at risk of or currently experiencing hypertensive disorders during pregnancy. It highlights the significance of early detection, close monitoring, and tailored care plans to optimize both maternal and fetal well-being. Nevertheless, certain concerns outlined below warrant additional clarification.

First, as described in the study^1^, the subjects of this research were women with hypertensive disorders during pregnancy, with 316 having hypertension. Clearly, for this subgroup of hypertensive patients, the absence of treatment strategies for hypertension is evident. Research^2^ that included 22 studies involving 4,464 participants showed a significant association between atenolol and an increased risk of preterm birth. The occurrence of severe hypertension significantly decreased after taking nifedipine and methyldopa, indicating a relationship between antihypertensive strategies and maternal-fetal outcomes. Moreover, another meta-analysis^3^ revealed that all frequently prescribed antihypertensive medications during pregnancy have the capacity to lower the risk of severe hypertension. Notably, labetalol stands out as it can also diminish the risk of proteinuria, preeclampsia, and fetal or neonatal mortality. The evidence mentioned above suggests that antihypertensive strategies are crucial for women with hypertensive disorders during pregnancy, which are related not only to maternal outcomes but also to perinatal outcomes. Nevertheless, it is worth highlighting that this study lacks comprehensive information regarding the specific antihypertensive strategies employed, which represents a critical aspect of necessitating further elucidation.

Second, it is essential to emphasize that the study considers cesarean section as one of the crucial outcome indicators. However, cesarean sections are not solely related to women with hypertensive disorders during pregnancy but they are also influenced by numerous other factors, notably labor induction. Research conducted by Wilson et al^4^ demonstrates that labor inductions were related to a higher chance of cesarean sections. Similarly, a study by Thorsell et al^5^ indicates that inducing labor results in a higher risk of emergency cesarean sections for both nulliparous and multiparous women, as compared to labor that starts spontaneously. It is crucial to be mindful of this elevated risk when considering labor induction. Therefore, considering the significant relationship between cesarean section and labor induction, it is imperative to provide information about labor induction for both groups (chronic gestational hypertension and preeclampsia/eclampsia groups). In the absence of information on labor induction, the high rate of cesarean section may be attributed to the relatively higher rate of labor induction rather than being solely a consequence of chronic gestational hypertension or preeclampsia/eclampsia.
